# Strategies for Improving Sustainability in the Development of High-Performance Styrenic Block Copolymers by Developing Blends with Cellulose Derivatives

**DOI:** 10.3390/polym16060856

**Published:** 2024-03-21

**Authors:** Erika Pajares, Josu Fernández Maestu, Irati Fernandez-de-Mendiola, Unai Silvan, Pedro Costa, Iker Agirrezabal-Telleria, Carmen R. Tubio, Sergio Corona-Galván, Senentxu Lanceros-Mendez

**Affiliations:** 1 BCMaterials, Basque Center for Materials, Applications and Nanostructures, UPV/EHU Science Park, 48940 Leioa, Spain; erikapajares@hotmail.com (E.P.); josu.fernandez@bcmaterials.net (J.F.M.); irati.fernandezdemendiola@ehu.eus (I.F.-d.-M.); unai.silvan@bcmaterials.net (U.S.); senentxu.lanceros@bcmaterials.net (S.L.-M.); 2 Sustainable Process Engineering Group, Department of Chemical and Environmental Engineering, University of the Basque Country (UPV/EHU), 48013 Bilbao, Spain; iker.aguirrezabal@ehu.eus; 3 Department of Cell Biology and Histology, Faculty of Medicine and Nursing, University of the Basque Country (UPV/EHU), 48940 Leioa, Spain; 4 IKERBASQUE, Basque Foundation for Science, 48009 Bilbao, Spain; 5 Physics Center of Minho and Porto Universities (CF-UM-UP) and LaPMET—Laboratory of Physics for Materials and Emergent Technologies, University of Minho, 4710-057 Braga, Portugal; pcosta@fisica.uminho.pt; 6 Dynasol, Titán 15, 9th Floor, 28045 Madrid, Spain; scoronag@repsol.com

**Keywords:** high-performance polymers, blends, styrenic block copolymer, thermoplastic elastomer, cellulose derivatives

## Abstract

Next-generation high-performance polymers require consideration as sustainable solutions. Here, to satisfy these criteria, we propose to combine high-performance styrenic block copolymers, a class of thermoplastic elastomer, with cellulose derivatives as a reinforcing agent with the aim of maintaining and/or improving structural and surface properties. A great advantage of the proposed blends is, besides their biocompatibility, a decrease in environmental impact due to blending with a natural polymer. Particularly, we focus on identifying the effect of different blending compounds and blend ratios on the morphological, structural, thermal, mechanical, electrical and cytotoxic characteristics of materials. This research provides, together with novel material formulations, practical guidelines for the design and fabrication of next-generation sustainable high-performance polymers.

## 1. Introduction

High-performance polymers (HPP) are essential candidates for a broad range of applications, including in the aerospace, automotive, electronic, medical, oil, gas and military fields [1,2,3,4]. Unlike conventional polymers, HPP are known to offer a superior range of properties, particularly mechanical, thermal and chemical, with the ability to tolerate and resist harsh environments and conditions, such as a corrosive environment and high pressure and temperature, among others [5,6]. Significant efforts have been successfully made to develop and enhance their properties, in particular by the synthesis of new formulations, blends and composites [7] as well as by developing advanced processing methods, including additive manufacturing technologies such as fused deposition modelling (FDM) and direct ink writing (DIW) [6,8,9].

Different kinds of HPP, including thermoplastics and thermosets have been developed. They include liquid crystalline polymers, fluoropolymers, epoxy resins, polyurethanes, and siloxanes, among others [10]. In particular, thermoplastic elastomers (TPEs) have received considerable attention because of their mechanical properties and processability. They consist of two phases (elastomeric or soft phase, and thermoplastic or hard phase), each one providing different features [11]. One of the most relevant TPEs is the styrene-b-(ethylene-co-butylene)-b-styrene (SEBS) copolymer [12,13]. Petroleum-based SEBS is obtained through the hydrogenation of the styrene-butylene-styrene (SBS) copolymer to remove the unsaturation present in polybutadiene; the latter, in turn, being obtained through the polymerization of two containing monomers (styrene and butadiene). SEBS is biocompatible, stable under thermal and oxidizing conditions, resistant to UV radiation, elastic and easy to process [12]. Such versatile features make SEBS very attractive for use in the automotive industry, footwear, adhesives, and sensors, as well as in medical devices [14,15,16,17].

Recent efforts have significantly improved specific performance parameters and functional properties, in particular by the development of SEBS-based composites and blends. As representative examples, the introduction of inorganic fillers ranging from clay [18], graphite [19], and carbon black [20] to multiwalled carbon nanotubes [16] allow the tuning of mechanical and electrical properties, depending on filler type and content. SEBS-based blends with organic compounds of synthetic origin, such as polyamide 6 (PA) [21,22] and polystyrene matrices [23], result in the improvement of impact strength, tensile strength and Young’s modulus, respectively. Further, SEBS has been also used as a compatibilizing agent in polymer blends. This is the case for polystyrene/high-density polyethylene [24], and polypropylene/polyamide 6 [25] blends, in which mechanical properties such as ductility were generally improved.

Despite considerable progress, an increase in environmental concerns and regulations has resulted in an urgent need for research and development of more sustainable polymer and, in particular, SEBS compounds. In particular, the production of SEBS is based on polymerization techniques, which are related to considerable energy consumption and greenhouse gas emissions, with negative environmental impacts. The growing demand for more sustainable products based on the principles of a circular economy requires materials to have a low environmental impact without compromising on the physicochemical properties needed for applications. In this regard, the use of organic agents of natural origin to meet sustainable demands is increasingly being considered. In the particular case of SEBS as a polymer matrix, it can be blended with biomass derivatives ranging from cellulose nanofibers [26] and cork [27] to pineapple leaf fibers [28]. However, more research is necessary to improve functionality and properties, and to broaden the potential range of applications.

Lignocellulosic biomass is particularly attractive as sustainable feedstock due to its renewability and abundance [29,30,31]. It consists of three main components: lignin, hemicellulose and cellulose. The latter is the most abundant component, with great potential as a biodegradable biopolymer. Cellulose is a polymer formed by glucose units with alternating amorphous and crystalline regions and containing hydrogen bonds [32]. In fact, the combination of SEBS and cellulose as a biopolymer has been addressed [26,33], with cellulose derivatives contributing to a mechanical reinforcing of the composites. In order to properly tune materials’ properties, the affinity between both matrices in the blends is critical, which is reliant on the chemical structure of the polymers.

**Figure 1:**
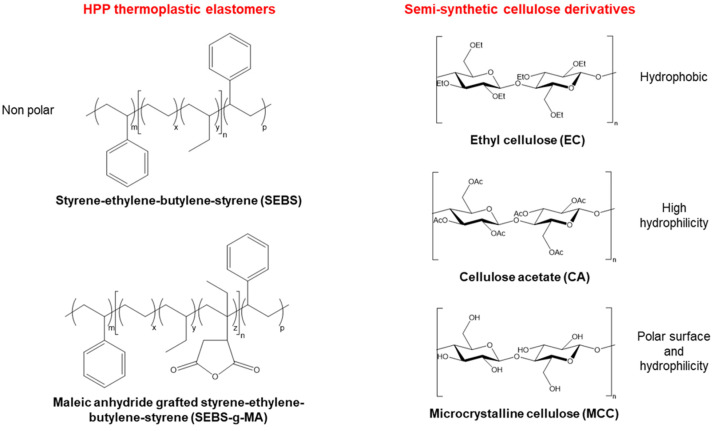
Chemical structures of SEBS, SEBS-g-MA, and cellulose derivatives.

In this work, we make use of a high-performance SEBS thermoplastic elastomer as well as SEBS functionalized with maleic anhydride (SEBS-g-MA) as the main components of a polymer blend in order to form a compatible and homogeneous or heterogeneous matrix with cellulose as the second component. The purpose of this study is to investigate the compatibility of cellulose derivatives with SEBS and SEBS-g-MA, and to evaluate the physical–chemical properties of the blends, in the scope of their potential applicability. Compatibility in the blend is based on exploring three cellulose derivatives: ethyl cellulose (EC), cellulose acetate (CA) and microcrystalline cellulose (MCC) [32,34,35]. These cellulose derivatives have been selected to reflect different hydrophobic/hydrophilic character and polarity (Figure 1). Notably, they have differences related to the number of hydroxyl groups reduced by etherification (EC), and esterification and transesterification (CA), whereas the degree of crystallinity is increased by purification and partial depolymerization (MCC). Using a solvent casting method, we successfully prepared several polymer blends with different ratios, and the morphological, structural, thermal, mechanical, electrical and cytotoxic properties were evaluated. The analyzed properties were used to investigate the effect of the addition of different types of cellulose into the non-functionalized and functionalized SEBS matrix, as well as to assess the effects of blending ratios. The results obtained from this work are expected to shed light on the design of more renewable polymer blends, leading to a transition to more sustainable high-performance polymers.

## 2. Materials and Methods

### 2.1. Materials

SEBS (15 wt% styrene content, 62% vinyl content, melt flow = 37.5 g/10 min (230 °C/2.16 kg)) and SEBS-g-MA (degree of functionalization 1.6 wt%, derived from a SEBS having 15 wt% styrene, 62% vinyl and melt flow = 10 g/10 min (230 °C/2.16 kg)) were provided by Dynasol Elastomers, Madrid, Spain. Ethyl cellulose powder (48–49.5% (W/W) ethyl basis), cellulose acetate powder (Mn ~30,000 g/mol, 39.8 wt% acetyl), and cellulose microcrystalline powder (particle size = 51 µm) were supplied by Sigma-Aldrich (St. Louis, MI, USA). Tetrahydrofuran (THF, GPC grade, Scharlab) was selected as a solvent. All the reagents and the solvent were used as received.

### 2.2. Sample Preparation

The films were produced by solvent casting, where blends of SEBS:cellulose derivatives, and blends of SEBS-g-MA:cellulose derivatives were produced in the blend ratios (by weight) of 100:0, 90:10, 80:20, and 70:30 (Table 1). The polymer to solvent ratio was 1:5 (wt:v). Specific solutions were prepared by dissolving the specific polymer in the THF solvent at ambient temperature and under magnetic stirring until complete dissolution. Then, the solutions were mixed under magnetic stirring at ambient temperature for 2 h. The mixture solutions were finally deposited using the doctor blade technique onto glass substrates, dried at room temperature for 12 h and films were peeled from the glass substrates. The obtained films show a thickness of around 50–70 µm, and a size of 2 × 5 cm^2^. The blends with MCC were prepared following the same procedure, with a previous ultrasonic step in an ultrasonic bath (Model ATM3L, ATU, Valencia, Spain) for around 2 h before mixing to ensure good dispersion of the MCC particles in the THF solvent.

### 2.3. Characterization

Cross-section scanning electronic microscopy (SEM) images were obtained using a Hitachi S-4800 microscope (Tokyo, Japan), using an accelerating voltage of 15 kV. Samples were coated with a 20 nm gold layer via sputtering deposition with a Polaron SC 502 sputter coater (Laughton, UK).

Fourier transform infrared spectroscopy (FTIR) spectra were recorded by using a Jasco FT/IR-4100 (Easton, MD, USA). in the attenuated total reflectance (ATR) mode from 600 to 4000 cm^−1^ with a resolution of 4 cm^−1^.

Contact angle measurements were carried out using a contact angle goniometer (OCA 15EC, Neurtek, Guipuzkoa, Spain).

Mechanical properties were evaluated by analyzing the tensile stress–strain curves of the samples. These tests were carried out using a Shimadzu AGS-J universal testing set up (Kyoto, Japan), with a load cell of 500 N until fracture at a test velocity of 3 mm/min. Stress–strain hysteresis cycles (up to 500 loading-unloading cycles) were recorded by applying three different maximum strains: 5%, 10%, and 30% of the initial sample length.

Dielectric measurements were carried out at room temperature using a Quadtech 1920 Precision LCR Meter (Sussex, WI, USA) in a 1 kHz–1 MHz frequency range with an applied voltage of 1 V. Five-millimeter diameter gold electrodes were sputtered with a 25 nm thin gold layer, using a Polaron SC502 (Quorum, Laughton, UK) sputter coater under nitrogen atmosphere. The capacity (*C*) and dielectric losses (tan *δ*) of the polymer blends prepared in the parallel plate configuration were obtained as a function of frequency. The real and imaginary part of the dielectric constant (*ε*′ and *ε*″) and the real component of the alternating current (AC) electrical conductivity ($\sigma_{AC}^{\prime }$) were obtained according to the following equations:

(1) $$\varepsilon \prime =\frac{C\cdot d}{\varepsilon_{0}\cdot A}$$

(2) $$tan\delta =\frac{\varepsilon^{\prime \prime }}{\varepsilon^{\prime }}$$

(3) $$\sigma_{AC}^{\prime }=\varepsilon_{0}\cdot \omega \cdot \varepsilon^{\prime \prime }$$

where *A* indicates the area, *d* is the sample thickness, $\varepsilon_{0}$ (8.85 × 10^−12^ F/m) is the permittivity of free space, and *w* = 2·π·*f* is the angular frequency.

The cytotoxicity of the samples was evaluated using an extract exposure test. Briefly, mouse embryonic fibroblasts (MEFs) were seeded in 24-well plates and cultured in complete culture medium composed of Dulbecco’s Modified Eagle Medium (DMEM) (Gibco), 10% fetal bovine serum and antibiotics (Pen/Strep) at 37 °C and 5% CO_2_. Samples of 1 cm^2^ of the SEBS and SEBS-g-MA-based blends were sterilized by UV irradiation for 30 min and incubated in 1 mL of complete culture medium for 24 h at 37 °C. Next, 600 µL of the extracts were added to the MEF cultures and allowed to exert their effect for an additional 24 h, before carrying out a Live/Dead cell viability assay (Thermofisher, Waltham, MA, USA). For the positive control, MEFs cultured in complete culture medium were used, and for the negative control, cells were permeabilized using cold 100% methanol. Fluorescence intensity for Calcein (live cells) and Ethidium homodimer (dead cells) was measured using a plate reader (Tecan, Switzerland). Each material was analyzed in triplicate. MEF images were taken using a Nikon Eclipse Ti-S/L100 inverted fluorescence microscope (Melville, NY, USA).

## 3. Results and Discussion

### 3.1. Morphological and Chemical and Surface Properties

**Figure 2:**
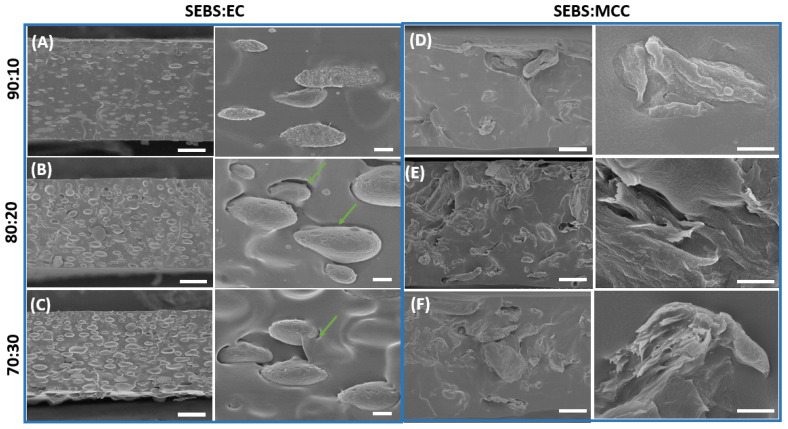
Cross-sectional SEM images of the SEBS-based blends at low magnification (left, 30 µm scale bar) and high magnification (right, 1 µm scale bar): (**A**) SEBS90:EC10, (**B**) SEBS80:EC20, (**C**) SEBS70:EC30, (**D**) SEBS90:MCC10, (**E**) SEBS80:MCC20, and (**F**) SEBS70:MCC30. The green arrows indicate voids at the different materials’ interfaces.

**Figure 3:**
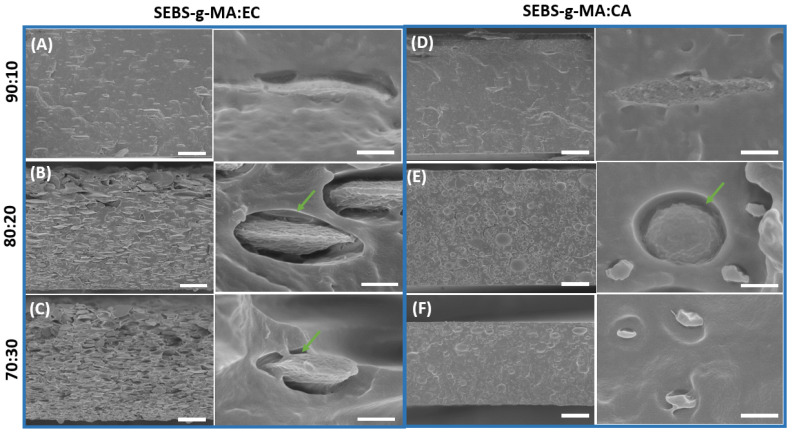
Cross-sectional SEM images of the SEBS-g-MA-based blends at low magnification (left, 30 µm scale bar) and high magnification (right, 1 µm scale bar): (**A**) SEBS-g-MA90:EC10, (**B**) SEBS-g-MA80:EC20, (**C**) SEBS-g-MA70:EC30, (**D**) SEBS-g-MA90:CA10, (**E**) SEBS-g-MA80:CA20, and (**F**) SEBS-g-MA70:CA30. The green arrows indicate voids at the different materials’ interfaces.

The performance of polymer blends is highly dependent on the miscibility of polymers, requiring a suitable morphological analysis. Factors such as the nature of both materials and blend composition are essential to determine miscibility. We tested combinations of styrenic block copolymers (SEBS and SEBS-g-MA) and cellulose derivatives (CA, CE, MCC) at 100:0, 90:10, 80:20 and 70:30 weight ratios. The mechanical consistency of films is suitable for most of the combinations, but the SEBS:CA and SEBS-g-MA:MCC samples presented several problems related to ink mixing and peeling films. This is attributed to the significant differences in the polarity of the materials [36,37]. Representative cross-sectional SEM images of the obtained SEBS and SEBS-g-MA-based blends with different weight ratios are shown in Figure 2 and Figure 3, respectively. The neat SEBS and SEBS-g-MA matrices (Figure S1) have a smooth and compact morphology. Moreover, the incorporation of cellulose derivatives resulted in a phase separation process, where two distinct phases are clearly identified: the styrene-based thermoplastic elastomer (continuous polymer matrix) and the cellulose derivatives (dispersed phase). This is due to the immiscibility of both components mainly caused by their difference in polarity. Interestingly, oval-shaped EC agglomerates are found uniformly dispersed through the SEBS (Figure 2A–C) and SEBS-g-MA (Figure 3A–C) matrices, which are oriented with the plane in the direction in which the films are produced through the doctor blade technique. Voids (green arrows) also appear at the interface of the materials’ components as a result of phase separation processes during solvent evaporation in the casting technique and the interfacial interaction of the material components [38]. By comparison, EC shows a better adhesion to SEBS (Figure 2A–C) than to SEBS-g-MA (Figure 3A–C). On the other hand, the lack of homogeneity and the extended agglomerate presence in the blends formed by SEBS and MCC (Figure 2D–F) is a clear indication of their poor compatibility. CA presents a similar adhesion than EC in the SEBS-g-MA matrix (Figure 3), but with regular circular-shaped agglomerates. In particular, compatibility is a function of the relative polarity between the two components and is thus critical for obtaining a homogeneous hybrid phase. SEBS and the chemically modified cellulose derivatives by etherification (EC) and esterification (CA) allow tuning of the interfacial compatibility when compared to SEBS with MCC cellulose derivative.

On the other hand, as seen from the optical microscope images in Figure S2A, color changes were observed after the cellulose derivative addition in the neat SEBS and SEBS-g-MA samples. In particular, the SEBS and SEBS-g-MA samples exhibit a transparent appearance. Moreover, the optical microscopy images reveal that the surface of the SEBS70:EC30 blend remains transparent. However, it can be seen that the SEBS70:MCC30 blend has aggregates in the form of islands over the sample surface. In contrast, it was observed that the color of the SEBS-g-MA blends changed from transparent to white after the cellulose derivative addition.

**Figure 4:**
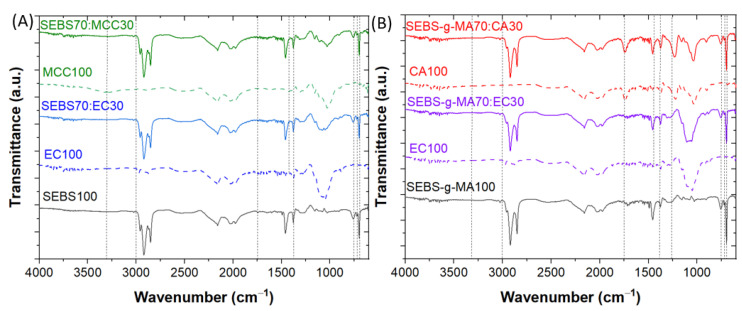
FTIR-ATR spectra of (**A**) SEBS and (**B**) SEBS-g-MA-based blends.

The surface of the hybrid materials has been also assessed by FTIR spectroscopy, as represented in Figure 4 for the samples with the highest cellulose contents. The FTIR spectra for SEBS and SEBS-g-MA differ due to the stretching vibrations bands of the carbonyl groups at 1769 and 1715 cm^−1^, respectively, as well as for the C–O–C stretching vibration bands at 1254 cm^−1^, arising from the maleic anhydride grafted onto the SEBS [39]. Styrene is related to the stretching vibration bands of the unsaturated bonds of the aliphatic groups at wavenumbers above 3000 cm^−1^, the C=C aromatic stretching at 1455–1600 cm^−1^ and the out-of-plane bending at 754 and 697 cm^−1^. Butadiene is reflected in the symmetric bending vibration band of the methyl group at 1380 cm^−1^, whereas ethylene is related to the C–H rocking vibration band at 718 cm^−1^. Both butadiene and ethylene contribute to CH_2_ group bending vibrations (1455 cm^−1^) [40,41,42]. The spectra of the neat cellulose derivatives (EC, MCC and CA) share several bands, the main differences being the hydroxyl group stretching at 3300 cm^−1^ observed in all samples, although with different intensities, because ethylation and acetylation in EC and CA strongly reduce this band [43]. Likewise, the common bands for EC and CA are related to the methylene and methyl group at 1455 and 1375 cm^−1^ [44,45], whereas the one corresponding just to CA is the carbonyl vibration at 1738 cm^−1^ [45].

By comparing the spectra of the neat compounds with those of the blends, the FTIR bands presented in Figure 4 demonstrate that there is no chemical interaction between the two materials as no new bands appear indicating the possible formation of new hydrogen or primary bonds.

**Figure 5:**
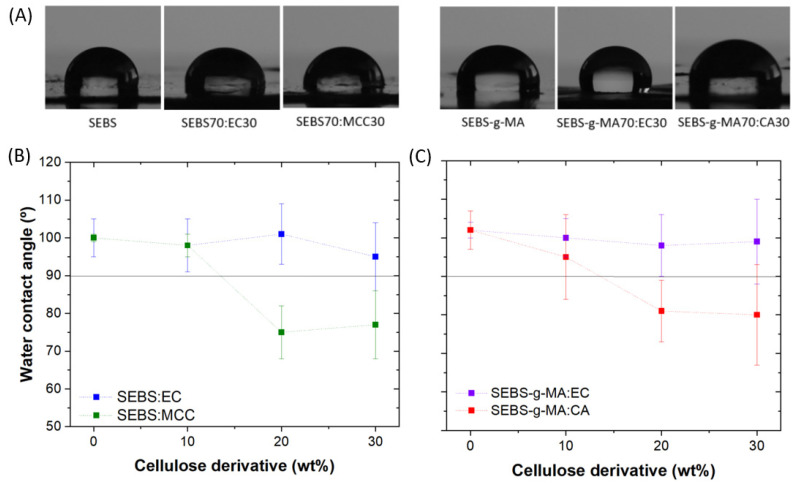
(**A**) Optical images of water droplets on the different blends. Water contact angle measurement of (**B**) SEBS and (**C**) SEBS-g-MA-based blends. The measurement was performed in three samples and the error bar represents the standard deviation.

On the other hand, we also tested the hydrophobic/hydrophilic surface characteristics of the samples (Figure 5). Neat SEBS films are hydrophobic with a contact angle of around 102°, whereas SEBS-g-MA shows a contact angle of 100°, highlighting the hydrophobic character of these polymers [46,47]. Regarding the effect of EC content within the polymer blend, no relevant variations have been observed. On the other hand, the inclusion of MCC leads to a reduction in the contact angle down to 77° for the sample with the higher filler contents, due to its hydrophilic character. Similarly, CA also leads to filler content surface variations of SEBS-g-MA-based blends, as reflected by the water contact angle around 80° obtained for the sample with 30 wt% CA content [48].

### 3.2. Mechanical Properties

**Figure 6:**
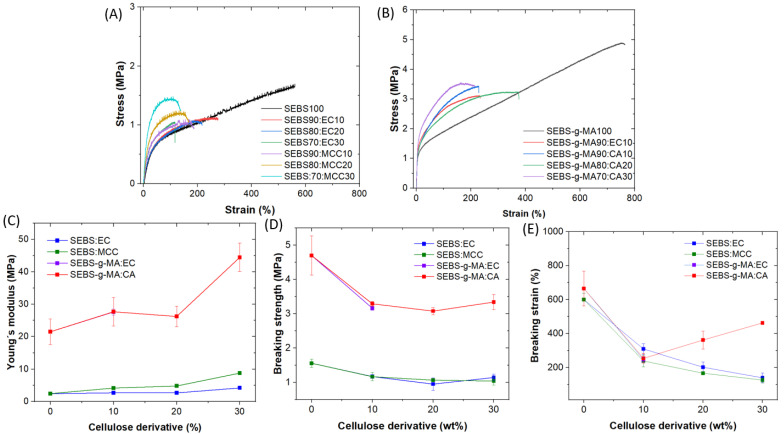
Stress–strain curves of (**A**) SEBS, and (**B**) SEBS-g-MA-based blends. Variation of (**C**) Young’s modulus, (**D**) breaking strength, and (**E**) breaking strain values with the cellulose content in the blends.

The stress–strain mechanical curves of the SEBS and SEBS-g-MA-based blends with varying cellulose derivative contents are shown in Figure 6A,B, respectively. Note that the curves could not be compared with the SEBS-g-MA80:EC20 and SEBS-g-MA70:EC30 samples because tensile testing could not be conducted due to the fragility of the samples. Figure S2B shows a photograph of the SEBS-g-MA70:EC30 sample after being stretched. From the testing results, a strong variation in the mechanical characteristics of the blend with respect to the ones of the neat polymers can be observed. In particular, the elongation at break decreases significantly due the presence of cellulose derivatives in the blend. Thus, the addition of cellulose derivatives leads to a decrease in the elasticity and flexibility of the TPE. Figure 6C–E shows the dependence of the Young´s modulus and breaking tensile and strain of the blends as a function of cellulose contents. For the neat SEBS-g-MA, the Young´s modulus and the breaking tensile values are 21.48 MPa and 4.7 MPa, respectively, which are also much higher than that of neat SEBS with values of 2.44 MPa and 1.56 MPa, respectively. On the other hand, with the addition of cellulose derivatives, the Young´s modulus increases, whereas the tensile values decrease. Interestingly, the use of 30 wt% CA in combination with SEBS-g-MA results in the highest value for Young´s modulus (44.47 MPa). By comparison, among the different cellulose derivatives, the addition of EC in the SEBS and SEBS-g-MA-based blends caused just a slight variation in the Young´s modulus and tensile values.

In general, the variation in the results between the different compositions is caused by factors such as the interfacial interaction between the polymer matrix and the filler agent, or the degree of dispersion of the filler agent in the matrix due to low compatibility as a result of polarity differences. In this way, despite the non-polarity of the SEBS matrix, there is an increase in its polarity due to the modification through the maleic anhydride graft. Therefore, neat TPE polymers possess different properties, as reflected in the mechanical results. However, it is also worth noting that the polarity of the cellulose derivative causes changes in the blends, as can be seen in the SEM results. This is due to the modification of the cellulose and the replacement of hydroxyl groups by other functions, facilitating better adhesion and uniformity in hybrids of similar polarity. This can be observed in the previous SEM images of SEBS:EC and SEBS-g-MA:EC blends, which are more uniform than the blends with MCC and CA fillers. For this reason, the SEBS:EC blends maintain similar and better mechanical properties, in spite of the decrease in ductility caused by the reduction in elongation.

The data suggest that the presence of cellulose fillers results in less stress transfer between the matrix and the dispersed phase. In particular, the agglomerates act as a breaking point due to the defects at the interface caused by the reduced adhesion between the two components. Depending on the material application, it is evident that the blends of thermoplastic elastomer and renewable filler agent at low concentrations can serve as a replacement for the use of neat thermoplastic elastomer.

**Figure 7:**
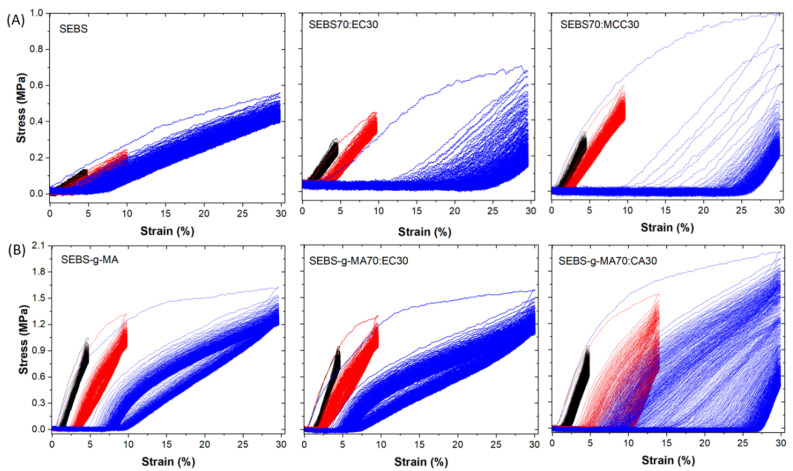
Loading–unloading mechanical tensile curves for (**A**) SEBS, and (**B**) SEBS-g-MA-based blends under 5% strain (black line), 10% strain (red line) and 30% strain (blue line).

A loading–unloading mechanical cyclic tensile test was then carried out to investigate the mechanical stability of the different composites. The cyclic tests were conducted under 5%, 10% and 30% applied strains for 500 cycles. Figure 7 shows the cyclic behavior of the neat styrenic block copolymers, and of the corresponding blends with the highest contents of cellulose derivatives. All samples show hysteretic behavior, indicative of the energy dissipation, and exhibit the Mullins effect, which is characterized by a decrease in the stress upon unloading compared to the stress upon loading at the same strain [49]. In addition, the maximum stress for a given strain of each cycle is higher for SEBS-g-MA blends than for SEBS blends, indicating a more flexible interface due to the presence of maleic polar groups. The addition of EC and MCC to the SEBS matrix (Figure 7A) shows a significant impact on the hysteresis loop, resulting in an increase in the loop, i.e., in the dissipated energy, the effect being more pronounced with the addition of MCC. Meanwhile, the incorporation of EC in SEBS-g-MA blends (Figure 7B) does not produce significant response differences, whereas the addition of CA causes an increase in the hysteresis loop for all evaluated strains.

### 3.3. Thermal Properties

**Figure 8:**
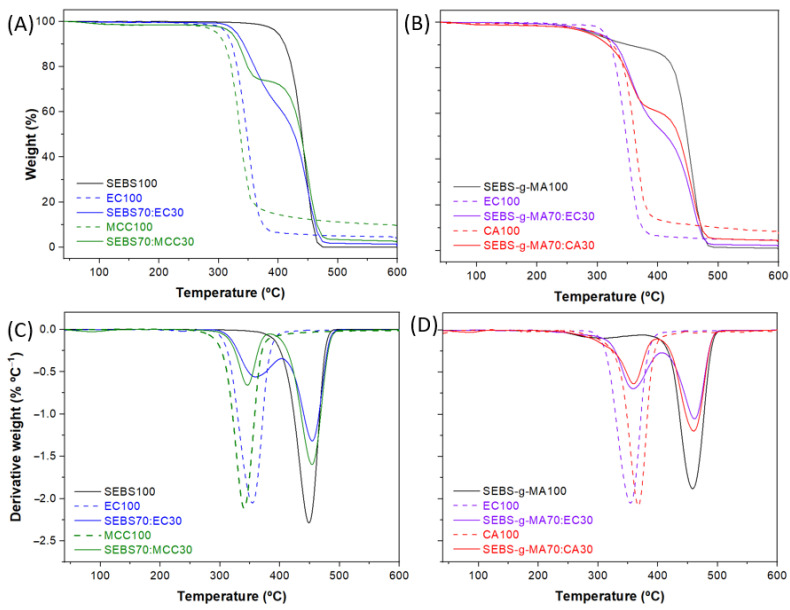
TGA and DTG thermograms of the (**A**,**B**) SEBS and (**C**,**D**) SEBS-g-MA-based blends.

Figure 8 shows the TGA curves for the SEBS and SEBS-g-MA-based blends. Also, the corresponding derivative thermogravimetry (DTG) curves are shown, where each peak determined the maximum rate of the degradation processes.

When comparing neat SEBS with the neat, functionalized SEBS-g-MA, the degradation of samples starts at higher temperatures, at a 10 wt% loss temperature (T10%) of 409 °C in the case of SEBS and at lower temperature (at 349 °C) in the case of SEBS-g-MA. Thus, the presence of maleic anhydride grafted onto the copolymer decreases the degradation temperature and thus the thermal stability. This effect is observed in many other polymers when they are grafted with MA, such as poly(hydroxybutyrate-co-hydroxyvalerate) (PHBV) [50] and poly(acrylamide/gelatin) hydrogels [51], among others. Moreover, it can be distinguished that SEBS is characterized by a single degradation stage in the range from 350 to 480 °C, whereas two stages are observed in SEBS-g-MA, one between 225 and 380 °C and the other between 380 and 500 °C. In the case of the SEBS-g-MA sample, the first step could relate to anhydride units of MA [52].

On the other hand, in the case of neat cellulose derivatives, a single degradation stage is observed. This stage and weight loss is attributed to the breakdown of the anhydroglucose polymeric chain, i.e., the breaking of the β-1,4-glycosidic bonds that hold the glucose units together, followed by the primary decomposition of volatile and dehydrated compounds. It corresponds to the processes of dehydration, depolymerization and decomposition of glysosyl units [53].

With respect to the composite samples, several stages of degradation can be distinguished, related to the composite composition. With regard to the SEBS:cellulose samples (Figure 8A,B), the first stage of degradation is attributed to cellulose (EC or MCC) and appears in the range from 300 to 370 °C, and the second stage is related to the degradation of the block copolymer and appears in the range from 370 to 490 °C. For this reason, the SEBS blends with cellulose derivatives present a decrease in thermal stability compared to the neat SEBS. This decrease is caused by the cellulose because, as described previously, the decomposition of the anhydroglucose polymer chain in the cellulose occurs at a lower temperature than in the copolymer. Several processes are involved, such as the breaking of the β-1,4-glycosidic bonds holding the glucose units together and then the primary decomposition of volatile and dehydrated compounds.

On the other hand, in the SEBS-g-MA-based blends (Figure 8C,D) prior to the decomposition of the anhydroglucose chain, a slight SEBS degradation occurs at 230 °C for SEBS-g-MA blends with cellulose derivatives. This is caused by the presence of anhydride units of MA, affecting the weight loss to a minor extent at such low content. The presence of the maleic anhydride graft in SEBS, however, decreases the thermal stability of the copolymer. Subsequently, the two main stages mentioned above (cellulose and copolymer degradation) occur after 230 °C. In addition, the substitution of hydroxyl groups in the cellulose by ethyl or acetate groups leads to a slight increase in thermal stability. Therefore, the SEBS:EC blend is thermally more stable than the SEBS:MCC, and the SEBS-g-MA:EC blend is somewhat more stable than the SEBS-g-MA:CA. The onset of CA degradation occurs earlier than that of EC, although the maximum degradation temperature is slightly higher in CA. By comparing the different polymer blends, all the maximum degradation temperatures lay ±10 °C compared to neat materials, confirming the immiscibility of the polymers. After the thermogravimetric analysis of the samples, the content of the final residue depends on the amount of added filler and its crystallinity.

### 3.4. Electrical Properties

**Figure 9:**
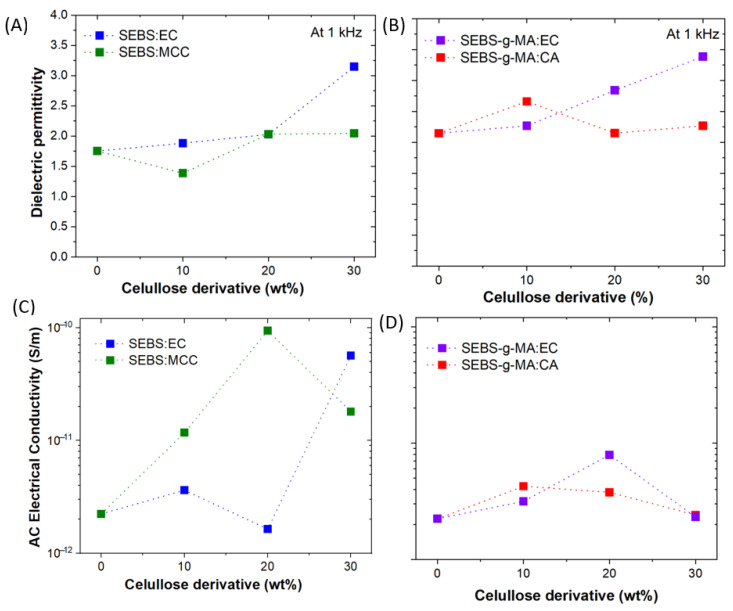
Dielectric permittivity values at 1 kHz for (**A**) SEBS and (**B**) SEBS-g-MA-based blends. Evolution of aAC electrical conductivity as a function of cellulose derivatives content for (**C**) SEBS and (**D**) SEBS-g-MA-based blends.

In order to study the potential of blends for electronic applications, the dielectric properties of the blends have been evaluated. The frequency dependence of the real dielectric permittivity (*ε*′) for the different SEBS (Figure S3A) and SEBS-g-MA (Figure S4A) blends shows a relative stable behavior over frequency variation. Meanwhile, the corresponding dependence of the dielectric loss (tan *δ*) on the frequency for SEBS (Figure S3B) and SEBS-g-MA (Figure S4B) blends shows similar behavior in both samples. The tan *δ* values are maintained below 0.15 over the measured frequency range. For comparison, the *ε*′ value at 1 kHz for SEBS and SEBS-g-MA-based blends with cellulose derivatives is shown in Figure 9A,B, respectively. Significantly, dielectric permittivity increases by a factor of two for the blends with the 30 wt% EC, when compared to the neat SEBS polymer (*ε*′ = 1.75) and neat SEBS-g-MA polymer (*ε*′ = 2.15). Finally, the ac electrical conductivity was calculated for the different samples (Figure 9C,D). An increase of nearly 2 orders of magnitude is obtained in the SEBS blend (Figure 9C) with the highest content of EC and MCC. It is in agreement with previous results on the effect of MCC that improves dielectric properties [54]. On the other hand, it is evidenced that the AC conductivity in the SEBS-g-MA-based blends (Figure 9D) remains stable with the EC and CA addition.

### 3.5. Indirect Cytotoxicity Analysis

**Figure 10:**
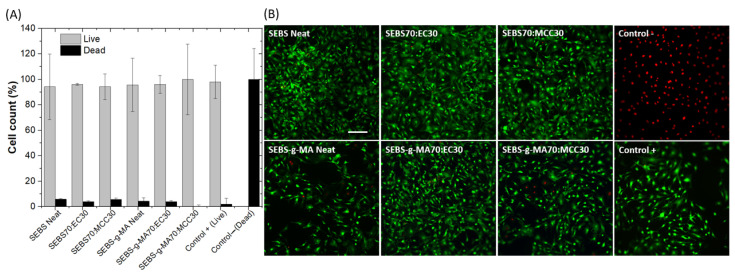
(**A**) All blend extracts display high biocompatibility as estimated using a Live/Dead assay. Data shown as average +/− SD. (**B**) Fluorescence images show a large number of live cells (green) in the cultures in contact with the material extracts with a residual number of dead cells (red). Scale bar represents 200 µm.

The biocompatibility of the SEBS and SEBS-g-MA-based blends was evaluated after an extract exposure test. For this, we incubated the materials in complete culture medium for 24 h and subsequently used it to grow mouse embryonic fibroblasts (MEFs). The obtained results show high biocompatibility of all analyzed materials with similar survival values to those of the positive control (Figure 10A,B). Thus, the results are consistent with the literature that indicate negligible cytotoxicity for SEBS samples [55], as well as cellulose derivatives [56].

## 4. Discussion

Cellulose derivatives up to 30 wt% have been explored as potential additives to improve the sustainability of high-performance styrenic block thermoplastic elastomer. The strategy implemented here explores styrenic block copolymers SEBS (no polar) and SEBS-g-MA (polar) and cellulose derivatives with a different polar surface area (PSA) from weaker polar EC (PSA = 134.53 Å), and MCC (PSA = 167.53 Å) to moderately polar CA (PSA = 238.09 Å). SEBS, SEBS-g-MA and EC are hydrophobic, differing from the hydrophilic CA and MCC. Blends are produced by solvent casting, leading to composites with different mechanical and electrical characteristics (Table 2), which can be taken advantage of for different applications. The use of EC in SEBS and SEBS-g-MA blends preserves the processability and wettability of styrene copolymers and increases the dielectric response. Also, the incorporation of EC leads to a decrease in the mechanical properties, such as the tensile strength, modulus and elongation at break, without impacting significantly on the dissipative properties of neat styrene block copolymers. This is an interesting result, considering the potential applications of the styrenic block copolymers, where the mechanical properties are essential. Therefore, the role of the hydrophobic character of these three polymers is a critical factor in controlling compatibility.

On the other hand, the use of hydrophilic MCC and CA polymers results in more negative effects on the physicochemical properties of the blends. Specifically, crack-free films can only be obtained when using polymers with similar polarity (SEBS:MCC, SEBS-g-MA:CA). However, the other combinations of SEBS:CA and SEBS-g-MA:MCC result in broken films, which reveals the importance of wettability and polarity. In addition, these cellulose polymers do not provide significant changes in dielectric properties, when compared to neat styrenic block copolymers. Instead, the wettability is modified, with respect to the pristine polymer, in all compositions.

## 5. Conclusions

In summary, we report the use of cellulose derivatives in the formation of styrenic block copolymer-based blends to improve the sustainability of high-performance polymers. A series of experimental tests were conducted to assess the influence of different cellulose derivatives and contents on the physicochemical and structural characteristics of styrene-based blends. The results show that the morphology of blends is highly dependent on the cellulose derivative type and content. The SEM results revealed that the presence of EC causes the formation of regular oval-shape aggregates, where the interfacial adhesion with SEBS is higher than with SEBS-g-MA, also depending on the polarity of the specific cellulose derivative. On the other hand, blending with cellulose derivatives leads to a decrease in tensile properties, such as tensile strength, and elongation at break, compared to the neat styrenic block copolymers. However, the presence of EC showed a positive effect on the tensile energy dissipation, without significant changes. Furthermore, the analysis of FTIR showed no interaction among the components of blends, whereas thermal testing shows that the cellulose derivatives decrease thermal stability compared to the neat styrene copolymers. Meanwhile, the dielectric permittivity of both SEBS and SEBS-g-MA were enhanced by a factor of two upon addition of 30 wt% EC.

Finally, all SEBS and SEBS-g-MA-based blends showed no cytotoxicity after 24 h for MEFs cells. Based on the above results, it can be concluded that EC at different concentrations can effectively be used in blends with SEBS type materials in order to tune mechanical, thermal and electrical properties, representing a promising pathway for the preparation of more sustainable high-performance styrenic block copolymers-based blends.

## Figures and Tables

**Table 1:** Blend formulation according to weight percentage (wt%) of each component.

Identification Sample	SEBS (wt%)	SEBS-g-MA (wt%)	EC (wt%)	CA (wt%)	MCC (wt%)
SEBS100	100	0	0	0	0
SEBS90:EC10	90	0	10	0	0
SEBS80:EC20	80	0	20	0	0
SEBS70:EC30	70	0	30	0	0
SEBS90:MCC10	90	0	0	0	10
SEBS80:MCC20	80	0	0	0	20
SEBS70:MCC30	70	0	0	0	30
SEBS-g-MA100	0	100	0	0	0
SEBS-g-MA90:EC10	0	90	10	0	0
SEBS-g-MA80:EC20	0	80	20	0	0
SEBS-g-MA70:EC30	0	70	30	0	0
SEBS-g-MA90:CA10	0	90	0	10	0
SEBS-g-MA80:CA20	0	80	0	20	0
SEBS-g-MA70:CA30	0	70	0	30	0

**Table 2:** Comparison between the main characteristics of the different blends.

Blends	Morphology	Mechanical	Wettability	Dielectric	Citotoxicity
SEBS:EC	Regular oval-shaped aggregates		Hydrophobic	Enhanced	Not cytotoxic
SEBS:CA	Not applicable	Not applicable	Not applicable	Not applicable	Not applicable
SEBS:MCC	Irregularly-shaped aggregates		Hydrophilic	Preserved	Not cytotoxic
SEBS-g-MA:EC	Regular oval-shaped aggregates	Preserved tensile energy dissipation	Hydrophobic	Enhanced	Not cytotoxic
SEBS-g-MA:CA	Regular circular-shaped aggregates		Hydrophilic	Preserved	Not cytotoxic
SEBS-g-MA:MCC	Not applicable	Not applicable	Not applicable	Not applicable	Not applicable

## Data Availability

Data available on request due to restrictions of privacy. The data presented in this study are available on request from the corresponding author. The data are not publicly available due to restrictions of privacy.

## Supplementary Materials

The following supporting information can be downloaded at: https://www.mdpi.com/article/10.3390/polym16060856/s1, Figure S1: Cross-sectional SEM images of SEBS and SEBS-g-MA samples at low magnification (left, 30 µm scale bar) and high magnification (right, 5 µm scale bar) Figure S2: (A) Optical microscopy images for the SEBS and SEBS-g-MA based blend films with different cellulose derivatives. Scale bar: 500 µm. (B) Photograph of stretchable SEBS-g-MA70:EC30 blend film. Figure S3: (A) Dielectric permittivity, and (B) dielectric loss as a function of frequency for SEBS based blends with EC and MCC fillers. Figure S4: (A) Dielectric permittivity, and (B) dielectric loss as a function of frequency for SEBS-g-MA based blends with EC and CA fillers.
