# Supplementary material for: Strategies for Improving Sustainability in the Development of High-Performance Styrenic Block Copolymers by Developing Blends with Cellulose Derivatives
Source: Polymers (Basel). 2024 Mar 21;16(6):856. doi: 10.3390/polym16060856 (PMC10975516; doi:10.3390/polym16060856)
Supplement: Supplementary file 1 [file polymers-16-00856-s001.zip › polymers-2902328-supplementary.pdf]

polymers

MDPI

Supplementary Materials

Strategies for Improving Sustainability in the Development of High-Performance Styrenic Block Copolymers by Developing Blends with Cellulose Derivatives

Erika Pajares [1,2], Josu Fernández Maestu [1], Irati Fernandez-de-Mendiola [1,3], Unai Silvan [1,4], Pedro Costa [5], Iker Agirrezabal-Telleria [2], Carmen R. Tubio [1,*], Sergio Corona-Galván [6] and Senentxu Lanceros-Mendez [1,4,5]

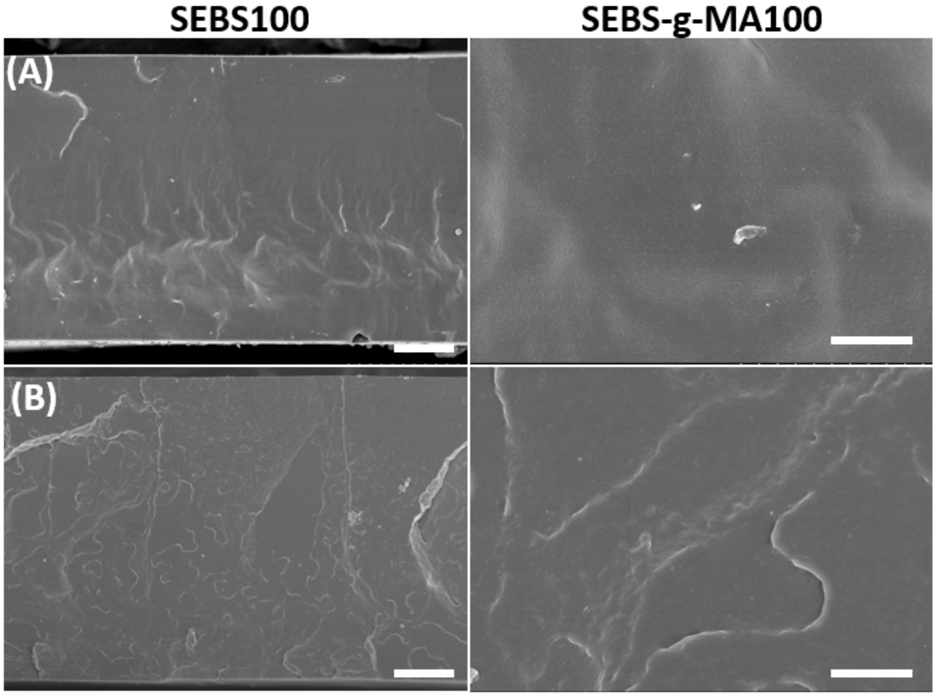

Figure S1. Cross-sectional SEM images of SEBS and SEBS-g-MA samples at low magnification (left, 30 µm scale bar) and high magnification (right, 5 µm scale bar).

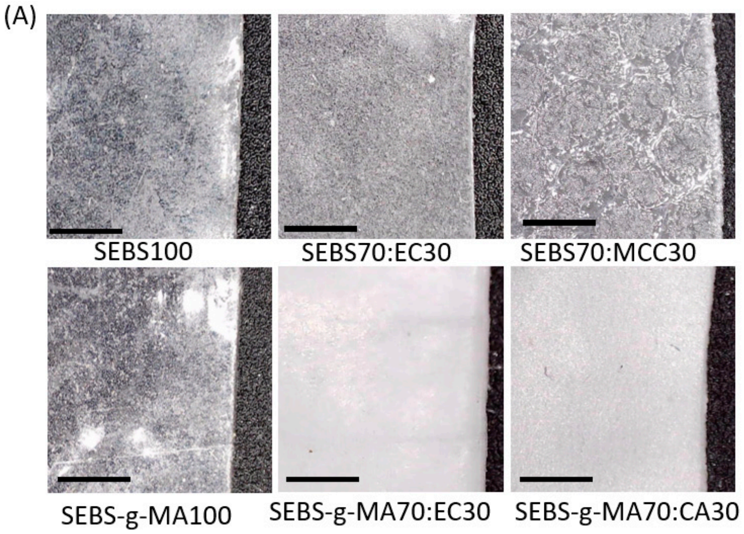

Figure S2. (A) Optical microscopy images for the SEBS and SEBS-g-MA based blend films with different cellulose derivatives. Scale bar: 500 μm. (B) Photograph of stretchable SEBS-g-MA70:EC30 blend film.

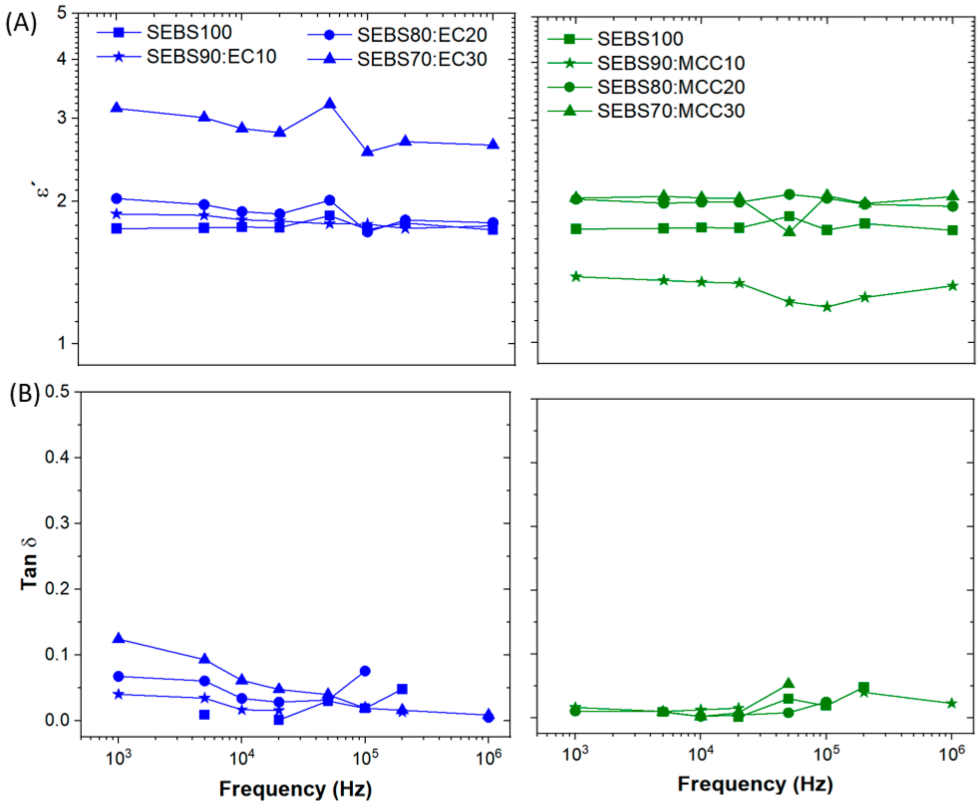

Figure S3. (A) Dielectric permittivity, and (B) dielectric loss as a function of frequency for SEBS based blends with EC and MCC fillers.

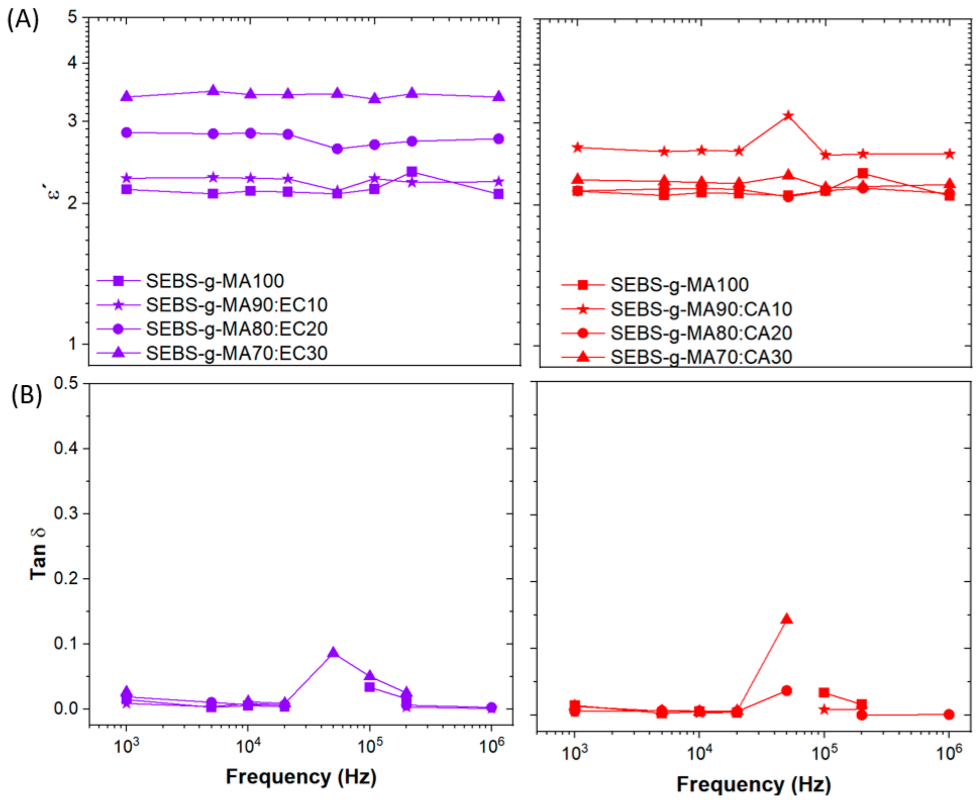

Figure S4. (A) Dielectric permittivity, and (B) dielectric loss as a function of frequency for SEBS-g-MA based blends with EC and CA fillers.
